# Improving Anti-listeria Activity of Thymol Emulsions by Adding Lauric Acid

**DOI:** 10.3389/fnut.2022.859293

**Published:** 2022-04-08

**Authors:** Qizhen Cai, Yun Zhang, Xiaofeng Fang, Suyun Lin, Zhirong He, Shengfeng Peng, Wei Liu

**Affiliations:** ^1^State Key Laboratory of Food Science and Technology, Nanchang University, Nanchang, China; ^2^Jiangxi Key Laboratory of Natural Products and Functional Food, College of Food Science and Engineering, Jiangxi Agricultural University, Nanchang, China; ^3^Jiangxi Danxia Biol Technol Co., Ltd., Yingtan, China; ^4^National R&D Center for Freshwater Fish Processing, Jiangxi Normal University, Nanchang, China

**Keywords:** Thymol, lauric acid, emulsion, *Listeria monocytogenes*, antibacterial

## Abstract

In this study, a novel emulsion, thymol (Thy) and lauric acid (LA) emulsion (Thy/LA-Emulsion) was prepared by homogenizing eutectic solvent (Thy/LA mixture) and caseinate solution. The effects of different thymol and lauric acid mass ratio on the formation, stability, and antibacterial activity of emulsions were studied. Compared with thymol alone, adding lauric acid (25, 50, and 75%) could enhance the antibacterial efficacy of the emulsions. Among them, Thy/LA_25%_-Emulsion could be stored at room temperature for a month without the increase of particle size, indicating that the addition of LA had not impacted the stability of emulsions. Meanwhile, Thy/LA_25%_-Emulsion exhibited a greater inhibition zone (3.06 ± 0.12 cm) and required a lower concentration (0.125 mg/mL) to completely inhibit the growth of *Listeria monocytogenes*. Consequently, Thy/LA_25%_-Emulsion demonstrated the best antibacterial activity and physicochemical stability due to its long-term storage stability. Our results suggest that Thy/LA_25%_-Emulsion may become a more functional natural antibacterial agent with greater commercial potential owing to its cheaper raw materials, simpler production processes, and better antibacterial effect in the food industry.

## Introduction

Approximately 600 million consumers get sick from the food contaminated by foodborne pathogens alone according to recognized outbreaks every year (1). Among the common foodborne pathogen infections, *Listeria monocytogenes* in bacteria has the highest fatality rate, which is up to 20–30% (2, 3). Consequently, it is essential to adopt effective measures to prevent foodborne illnesses caused by pathogens. Antibacterial agents with broad-spectrum and high-efficiency bactericidal effects are one of the hotspots of current research studies. However, the most commonly used kind of antibacterial agents, synthetic ones, still face a wide range of disadvantages, such as toxic residues, environmental pollution caused by their slow biodegradation, the high cost–benefit ratio, and the risk of microbial resistance (4, 5). Nowadays, consumers prefer natural antibacterial agents as substitutes for chemical preservatives to inhibit bacteria and the studies on developing natural antibacterial agents are still in great demand.

Essential oils (EOs), volatile odoriferous oils, one of the alternatives to chemical preservatives, are aromatic oily liquids originated from a variety of plants, which possess diverse properties, including antibacterial, antifungal, antiviral, anti-inflammatory, antioxidant, and insecticidal activity (6–10). Earlier, EOs were acquainted with potential natural antimicrobial agents and were recommended as “natural food additives” in food preservation. Nowadays, EOs are used as preservatives in the food industry to extend the shelf life of food (11). Thymol (2-isopropyl-5-methylphenol), a natural essential oil and phenolic compound, is a component derived from some medicinal plants, such as *Thymus*, *Origanum*, and *Coridithymus* (12). Thymol (Thy) has been proved to display considerable antibacterial activity against various bacteria and yeasts by disrupting bacterial membrane, leading to bacterial lysis and leakage of components inside microbial cells, resulting in cell death (13). Therefore, Thy was selected to develop an antibacterial agent in this study. Nevertheless, the utilization of Eos, especially Thy, in the food industry is partially limited owing to their poor solubility (14, 15) and instability (16) when exposed to light, oxygen, high temperature, and moisture, which might contribute to the degradation of EOs during the processing, transportation, storage, and consumption, or even a risk of forming toxic derivatives (17). Another reason that hinders the extensive utilization of EOs is that the antibacterial effect of a single kind of EOs is usually limited, and when sufficient amounts are added to exert potent antibacterial effects, they can affect food quality and lead to negative sensory effects (18).

To reduce the concentration of EOs without compromising their antibacterial ability, several synergies of diverse antibacterial compounds with EOs has been widely discussed, such as the synergistic effects of various EOs (19, 20), EOs and antibiotics (12, 21, 22), EOs and other antimicrobial agents [drugs (23), medium-chain fatty acids (MCFs) (24), polyphenols (25), etc.]. Among them, the MCFs are saturated fatty acids with 6–12 carbon atoms, including octanoic acid, capric acid, and lauric acid, which exist in nature in the form of medium-chain triglycerides, mainly in breast milk, milk and its products, coconut oil and palm oil, and little in other natural foods (26). Accompanied with antibacterial ability, MCFs have been demonstrated to restrain diverse foodborne pathogens, including *Escherichia coli*, *Salmonella*, and *Staphylococcus aureus* (27–29). According to a previous study (24), the synergistic activity of MCFs and EOs can not only enhance their antibacterial effect, but also lessen its unique odor and irritation by replacing a portion of EOs, and meanwhile minimize the loss of nutrients and quality of food by decreasing the number of antibacterial agents. Moreover, it is more in line with the prevailing market in terms of economic benefits because of the relatively lower price of MCFs. Nevertheless, the problems of poor solubility and instability of EOs remain unsolved by synergy with MCFs.

Several encapsulation systems (30, 31) have been developed to conquer the problem, such as liposomes, polymer particles, solid lipid nanoparticles, cyclodextrin, emulsions, and nanofibers. Using emulsions to encapsulate EOs is one of the feasible ways to widen their application, where the emulsions are claimed to be able to control release, target transport, and improve the solubility and stability of EOs (32, 33). In addition, emulsions can offer high drug-loading efficacy, which fits well with the prevailing market demand as the number of active substances in the antibacterial delivery system should be maximized. To the best of our knowledge, there are no previous reports concerning the influence of lauric acid (LA) on the formation, stability, and antibacterial activity of Thymol-based emulsions.

In this study, we attempted to resolve this dilemma by developing a novel emulsion, that is, by homogenizing the Thymol/Lauric acid (Thy/LA) solutions with caseinate solutions. And the optimal ratio of Thy/LA and their optimal proportion in the oil phase were selected. Finally, the impact of different mass Thy/LA ratios on emulsion stability and antibacterial effect was evaluated.

## Materials and Methods

### Materials

Thymol (98%) and lauric acid (98%) were purchased from Aladdin Biochemical Technology Co., Ltd (Shanghai, China). Sodium caseinate (NaCS) was provided by Sigma Chemical Company (St. Louis, MO, United States). Yeast extract and tryptone were donated by Oxoid (Beijing, China). Agar powder was obtained from Solar Science and Technology Company (Beijing, China). All other reagents used were of analytical grade.

### Preparation of Thy/LA-Emulsion

Briefly, 2 wt% NaCS solution was obtained by adding NaCS powder into phosphate-buffered solution (5 mM, pH 6.5) and then kept stirring for 4 h at room temperature. Thy and LA were mixed at different mass ratios (The ratios of LA are 0, 25, 50, 75, and 100 wt%.). Afterward, the mixtures were obtained by stirring at 65°C until a homogeneous liquid was formed. The final oil phases were prepared by mixing Thy/LA solutions and corn oil in various ratios (The ratios of corn oil are 0, 10, 20, 40, and 60 wt%.). The Thy/LA crude emulsions (Φ = 10%) were fabricated by stirring at 12,000 rpm for 3 min with a high shear dispersive machine (ULTRA TURRAX T18 Digital, IKA, Staufen, Germany). The final emulsion was obtained after passing through a microfluidizer (M-110EH30, Microfluidic Corp., Newton, MA, United States) at 70 MPa two times. In addition, the oil phase of the control group was prepared by mixing Thy and corn oil at corresponding mass ratios.

### Determination of Characterization of Thy/LA-Emulsion

The mean particle diameters (d_3_,_2_) and particle size distribution of the emulsions were measured by a laser diffraction instrument (Mastersizer 3000, Malvern Instruments Ltd., Worcestershire, United Kingdom) according to our previous method (34). The operating parameters used were as follows: lights obscuration was from 8 to 13%; the stirring speed was set as 3,500 rpm/s. Phosphate-buffered solution (5 mM, pH 6.5) was used throughout the test.

The ζ-potential of the emulsions was measured by using dynamic light scattering and electrophoresis (Nano ZS, Malvern Instruments, Worcestershire, United Kingdom) at 25°C. The emulsions were diluted 10-fold by using phosphate-buffered solution (5 mM, pH 6.5) to obtain an appropriate light intensity for reliable measurements.

### Determination of Thermal Property of Thy/LA-Emulsion

According to a previous method (35), differential scanning calorimetry (DSC X7000, Hitachi, Japan) was used to characterize the phase transitions during the melting process of Thy/LA mixed solution. By freeze-drying Thy/LA mixed solution, the powder was collected as the sample. The powder (1.8 mg) was weighed into an aluminum sample pan. The aluminum sample pan was heated from −10°C to 80°C at 10°C/min.

### Cryo-Scanning Electron Microscope

According to a previous method (36), the effect of LA addition on microstructure of Thy/LA-Emulsion was determined using cryo-scanning electron microscope (cryo-SEM) (HATACHI SU8010). The emulsions with conductive carbon glue were placed on a table, coated, frozen in liquid nitrogen slush, and then sublimated and gold-plated by using the cryogenic preparation and transmission system. The operating conditions of SEM were as follows: temperature, −140°C; accelerating voltage, 5 kV.

### Determination of *in vitro* Antibacterial Activity

#### Microorganisms

Two kinds of typical foodborne gram-negative bacteria [Escherichia *coli*, (10003, *E. coli)* and *Salmonella enterica* subsp. *enterica serovar Typhimurium* (22956, *S. Typhimurium*)] and two kinds of typical foodborne gram-positive bacteria [*Staphylococcus aureus* (21600, *S. aureus*) and *Listeria monocytogenes* (21635, *L. monocytogenes*)] were used to evaluate the antibacterial activity of the emulsions. The stock cultures were transferred 50 μl into 5 ml sterile Luria–Bertani broth (LB), which were revived at 37°C for 24 h to 10^–9^ cfu/ml. The cultures were diluted to 10^–4^ to 10^–6^ cfu/ml before use.

#### Determination of Minimum Inhibitory Concentrations and Minimum Bactericidal Concentrations

The minimum inhibitory concentrations (MICs) and minimum bactericidal concentrations (MBCs) of all Emulsions and Thy/LA mixed solution were determined by 96-well plate microdilution method based on a previous method (37). The different proportions of emulsion were diluted to 0.016–2 mg/ml in sterile LB, and Thy/LA mixed solutions were diluted to 0.0078–1 mg/ml in sterile LB quickly after heating, which were all prepared in a 96-well plate by an identical twofold serial dilution. Then 100 μl bacterial inoculum was added to each well, and the 96-well plates were incubated at 37°C for 24 h. The MIC was defined as the lowest concentration of an emulsion that inhibits the visible growth of bacteria. To determine MBC, 100 μl of culture broth with invisible growth was taken from each well and transferred to Luria–Bertani agar plate, and then incubated at 37°C for 48 h. The MBC was defined as the lowest concentration that bacteria did not grow at all on the agar surface.

#### Determination of the Zone of Inhibition

The zones of inhibition of all emulsions were measured by the Oxford cup method (38). Initially, 20 ml Luria–Bertani agar was poured into a 90-mm sterile Petri dish. After solidification, the diluted test strains (1 ml) were transferred into the agar surface and distributed evenly over the agar surface by a sterile bent glass rod. Then, 100 μl emulsion was taken and transferred into the sterilized Oxford cup (6 mm) that was located in the center of the dish. After standing for 5 min, the dish was incubated at 37°C for 48 h. The zone of inhibition (mm) was measured by a Vernier micrometer.

#### Determination of Growth Curve

To study the growth curves of all emulsions, the aerobic plate count was employed (39). Overnight test strains were diluted and transferred into sterile LB in sterile centrifuge tubes. Afterward, the emulsion was added to the tube at concentrations of 0, 2 MBC, MBC, 1/2 MBC, and 1/4 MBC. After incubating at 37°C, the mixtures were taken and transferred into the agar surface at 0, 4, 8, 12, and 24 h, respectively. After incubating at 37°C for 24 h, the colony number was calculated to draw the growth curve of Thy/LA-Emulsion.

### Statistical Analysis

All experiments were repeated at least three times. The mean and standard deviation were analyzed by statistical analysis software (SSPS, version 17.0). Statistical differences between experiments were detected by the least significant difference test (*p* < 0.05).

## Results and Discussion

### The Characterization and Antibacterial Activity of Thymol/Lauric Acid Mixture

**FIGURE 1:**
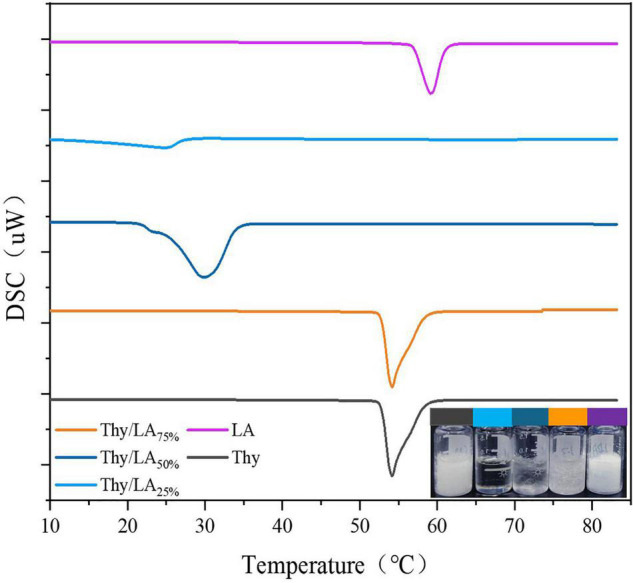
Differential scanning calorimetry (DSC) thermograms of thymol/lauric mixture (Thy/LA) with different mass ratios and the appearance of thymol/lauric acid mixture with different mass ratios at room temperature. All line colors correspond to the samples. *n* = 3.

Initially, the effect of Thy/LA mass ratio (The ratios of LA are 0, 25, 50, 75, and 100%.) on appearance, thermal properties by using differential scanning calorimetry (DSC) and antibacterial activity of Thy/LA was evaluated. The appearance of Thy/LA at different mass ratios is displayed in Figure 1. The state of mixtures depended on different mass Thy/LA ratios at room temperature, where Thy/LA_25%_ was a clear and transparent liquid and Thy/LA_50%_ and Thy/LA_75%_ were solid-like. These results are consistent with DSC thermograms exhibited in Figure 1. All mixtures displayed a single endothermic melting peak, and their melting point decreased as the content of LA decreased, which was lower than LA alone or Thy alone. This suggests that Thy and LA mutually inhibit crystallization, thus reducing the melting point of the mixed system, indicating that they have formed an eutectic solution rather than a simple eutectic mixture (40).

The antibacterial effects of Thy and LA with different proportions are illustrated in Table 1. At a concentration of less than 1 mg/ml, LA using alone did not show any antibacterial effect on four bacteria, while Thy exhibited a strong antibacterial effect on them, indicating that Thy had a better antibacterial effect than LA, and Thy was the principal antibacterial agent of the mixture. Compared with two Gram-negative bacteria, Thy showed a stronger antibacterial effect on two Gram-positive bacteria, among which the antibacterial effect against *L. monocytogenes* was best, so did Thy/LA. Moreover, the antibacterial effect against *L. monocytogenes* of Thy/LA with different proportions was comparable or better than those of LA and Thy alone. The MIC values for Thy/LA_25%_ (0.0625 mg/ml) and Thy/LA_50%_ (0.0625 mg/ml) were lower than LA (>1 mg/ml) and Thy (0.25 mg/ml) alone. The MBC values for Thy/LA_25%_ (0.25 mg/ml) and Thy/LA_50%_ (0.25 mg/ml) were lower than LA (>1 mg/ml) and Thy (0.5 mg/ml) alone, which were equivalent to a quarter of Thy alone, indicating that Thy/LA_25%_ and Thy/LA_50%_ demonstrated more effects against *L. monocytogenes*. This result ties well with a previous study (24) wherein Thy and LA exhibited stronger antibacterial effects than Thy or LA alone in certain proportions. According to previous studies (41, 42). Thy, as a hydrophobic substance, easily interacts with the phospholipid bilayers of bacteria to increase membrane permeability, which will lead to the leakage of components inside microbial cells, resulting in cell death. LA is also an amphiphilic substance, which can damage the cell membranes of Gram-positive bacterial (43). A possible explanation might be that the antibacterial effect of LA and Thy is not a simple superposition, but a synergistic effect (24), which may be because it acts on different sites of the cell membrane to increase membrane permeability, enhancing the antibacterial effect.

**TABLE 1:** The minimum inhibitory concentration (MIC) and minimum bactericidal concentration (MBC) of different mass thymol/lauric acid ratios against four foodborne pathogens *n* = 3.

Bacteria		Thy	Thy/LA_25%_	Thy/LA_50%_	Thy/LA_75%_	LA
*Listeria monocytogenes*	MIC(mg/mL)	0.25	0.0625	0.0625	0.25	–
	MBC(mg/mL)	0.5	0.125	0.125	0.5	–
*Escherichia coli*	MIC(mg/mL)	1	1	1	–	–
	MBC(mg/mL)	1	1	1	–	–
*Staphylococcus aureus*	MIC(mg/mL)	0.5	0.25	0.5	0.5	1
	MBC(mg/mL)	1	0.5	0.5	1	–
*Salmonella*	MIC(mg/mL)	1	0.5	0.5	1	–
	MBC(mg/mL)	1	1	0.5	–	–

*“–” illustrates that MIC or MBC are above 1 mg/ml.*

Owing to the remarkable antibacterial effect, LA with the ratio of 25 and 50% was selected to perform the following experiments, and *L. monocytogenes* was also selected to detect the bacteriostatic effect of the samples.

### Preparation, Characterization, and Stability of Thy/LA-Emulsion

**FIGURE 2:**
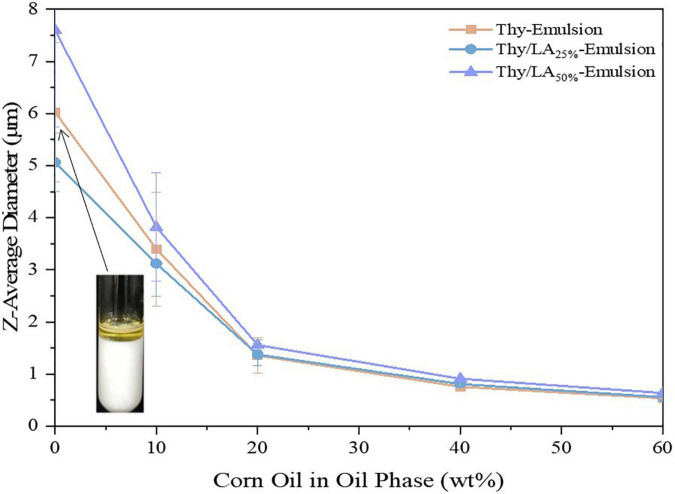
Effect of corn oil concentration (0–60 wt%) on the mean particle sizes of Thy/LA_25%_-Emulsion, Thy/LA_50%_-Emulsion, and Thy-Emulsion. The photo in the graph shows that Thy-Emulsion appeared oil separation (0% corn oil). *n* = 6.

The impact of the proportion Thy/LA in the oil phase (The ratios of LA are 0, 25, 50, 75, and 100 wt%.) on the mean particle diameter, stability, and microstructure by using cryo-SEM of the emulsions was evaluated. According to previous studies (32, 44), EOs are susceptible to Ostwald ripening (OR) causing instability in emulsions. OR is a common phenomenon in EO emulsions, based on the diffusion of components of dispersed phase from smaller to larger droplets through a continuous phase, leading to droplet growth, creaming, and oiling off. In several feasible solutions (37, 45), the simplest and most effective measure was to modify the oil composition by incorporating ripening inhibitors (corn oil) before homogenization to inhibit the OR. To increase the stability of the emulsion, corn oil was added to the oil phase to prepare emulsion, and the impact of LA on the physical stability of thymol-based emulsion was also studied. First, a series of emulsions (Φ = 10%) with different mass Thy/LA or Thy and corn oil ratios were prepared [corn oil (%):Thy/LA (%) or Thy alone (%) = 0:100, 10:90, 20:80, 40:60, 60:40]. After homogenization, the emulsions were stored for 3 days at room temperature, and their mean particle sizes were measured in Figure 2. A downward trend can be seen, where the mean particle size of the samples decreased as a higher proportion of corn oil added in the oil phase. The particle size of the emulsion prepared by the oil phase using only Thy (0% corn oil) was large and the emulsion was highly unstable as oil separation occurred in the emulsion after 1-week storage (Figure 2). The phase separation happened in Thy/LA_25%_-Emulsion and Thy/LA_50%_-Emulsion as well. The results are in line with previous research studies (46). Chang et al. investigated the impact of cationic surfactants (lauryl arginate) on the physical properties and antibacterial efficacy of thyme oil nanoemulsions. It was also found that the emulsions were highly unstable and rapidly separated at higher thyme oil levels (≥80 wt%), but emulsions with better stability could be attained by incorporating a ripening inhibitor (corn oil) to the oil phase before homogenization. This illustrates that the addition of corn oil does increase the stability of emulsions by inhibiting OR. There was a steep reduction in mean particle size of 0–20 wt% corn oil and a relatively gentle reduction in mean particle size of 20–60 wt% corn oil of all emulsions, indicating that all emulsions were more stable when the quantity of corn oil between 20 and 60 wt% in the oil phase, and the stability of emulsion was unaffected by the addition of LA. Prior research (32) has shown that the more corn oil is added, the more the antibacterial efficacy of emulsions is reduced. All in all, a ratio [corn oil (%):Thy/LA (%) or Thy (%) = 20:80] was selected for storage research and antibacterial experiments.

**FIGURE 3:**
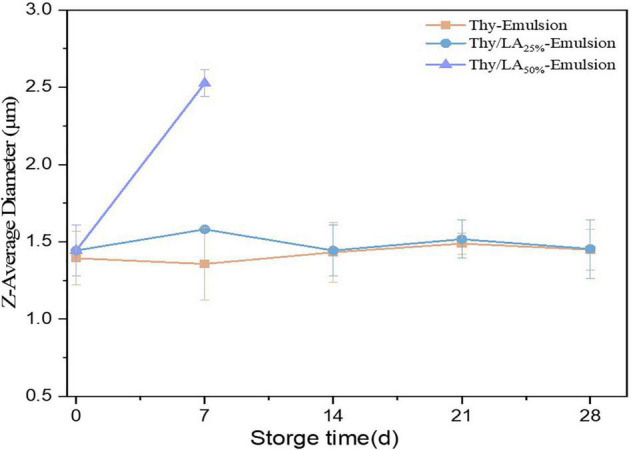
Changes of the mean particle sizes of Thy/LA_25%_-Emulsion, Thy/LA_50%_-Emulsion, and Thy-Emulsion during storage for 4 weeks. As solidification appeared on the surface of Thy/LA_50%_-Emulsion in the second week, particle size could not be measured. *n* = 3.

**FIGURE 4:**
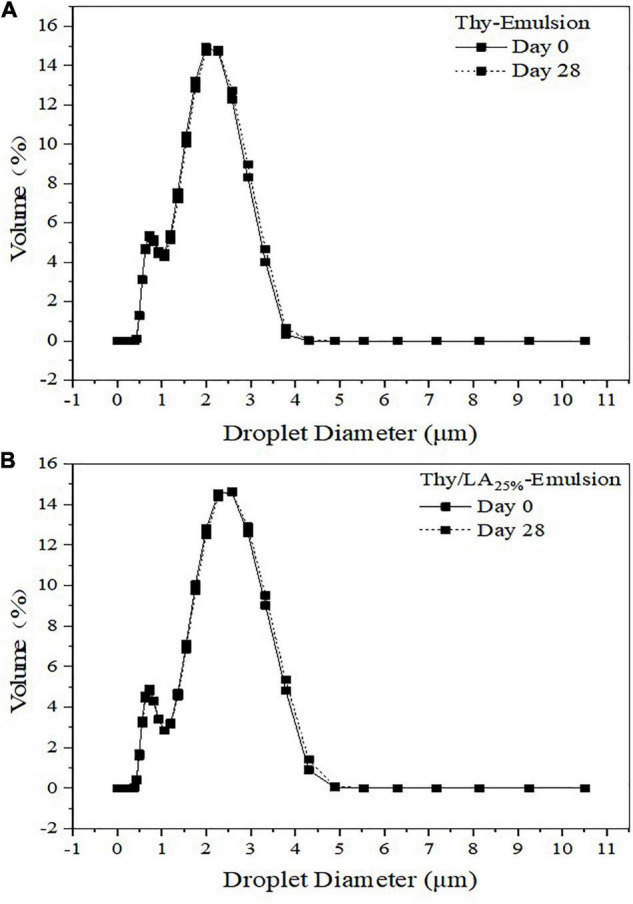
Droplet size distributions in the volume of Thy-Emulsion **(A)** and Thy/LA_25%_-Emulsion **(B)**. The solid line represents the 0th day, and the dashed line represents the 28th day. *n* = 3.

The mean particle size of three emulsions after 1-month storage at room temperature is shown in Figure 3. The mean particle size of emulsions is one of the critical factors to evaluate their physical stability (47). The mean particle size of Thy/LA_25%_-Emulsion and Thy-Emulsion did not appreciably change, illustrating that once an adequate quantity of ripening inhibitor is incorporated into the oil phase before homogenization, emulsions are highly stable against droplet growth over a period of time (48). These results are consistent with the volume fraction distribution of the particle size of Thy-Emulsion and Thy/LA_25%_-Emulsion exhibited in Figure 4. The volume fraction distribution of the particle size of Thy-Emulsion and Thy/LA_25%_-Emulsion were unimodal, and Thy-Emulsion and Thy/LA_25%_-Emulsion were stable after 28 days of storage. The mean particle size of Thy/LA_50%_-Emulsion increased from 1.44 ± 0.16 μm to 2.53 ± 0.08 μm after 1 week, and solidification appeared on the surface in the second week. Consequently, to ensure the storage stability of the emulsion, LA with the ratio of 25% was selected to perform the following experiments.

The ζ-Potential of an emulsion is generally related to the net surface electrical charge on the emulsion droplets and the stability of the emulsion (49, 50). The average ζ-potential values of Thy-Emulsion and Thy/LA_25%_-Emulsion were −33.47 ± 0.78 mV and −42.77 ± 0.81 mV, respectively. When pH was 6.5, which was higher than the isoelectric point of casein (pH 4.6), casein had a strong negative net charge (51). The emulsion droplets had a higher negative charge due to the absorption of casein at the oil–water (O/W) interface, so the ζ-Potentials of Thy-Emulsion and Thy/LA_25%_-Emulsion are negative. Moreover, with the addition of LA, the absolute value of zeta-potential of emulsion increased and the Thy/LA_25%_-Emulsion might have higher stability than Thy-Emulsion. It has been reported that emulsions with higher zeta-potential exerted stronger electrostatic interaction and greater repulsive forces between oil droplets, which could prevent aggregation and improve the stability of the system (52).

**FIGURE 5:**
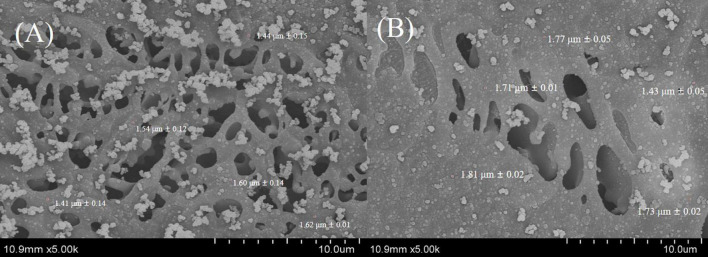
The cryo-SEM micrographs of the Thy-Emulsion **(A)** and Thy/LA_25%_-Emulsion **(B)**. *n* = 3.

Thy/LA_25%_-Emulsion and Thy-Emulsion were evaluated by cryo-SEM (Figure 5), which are consistent with the light scattering analysis of particle size. All the cryo-SEM images did exhibit that all emulsions with relatively small individual oil droplets (<2 μm) were evenly distributed throughout the samples, which also indicates that the stability of emulsions was not affected by the addition of LA.

### Antibacterial Activity of Thy/LA-Emulsion

**FIGURE 6:**
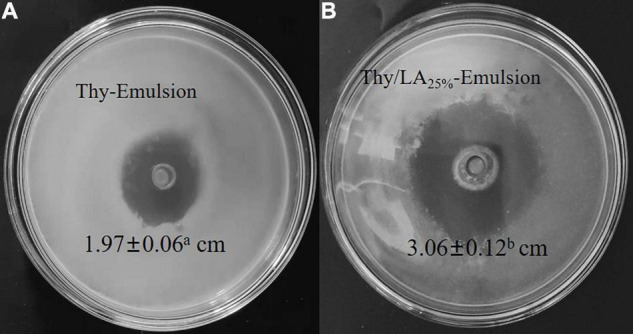
The Zone of Inhibition of Thy-Emulsion **(A)** and Thy/LA_25%_-Emulsion **(B)** against *Listeria monocytogenes*. *n* = 6.

**FIGURE 7:**
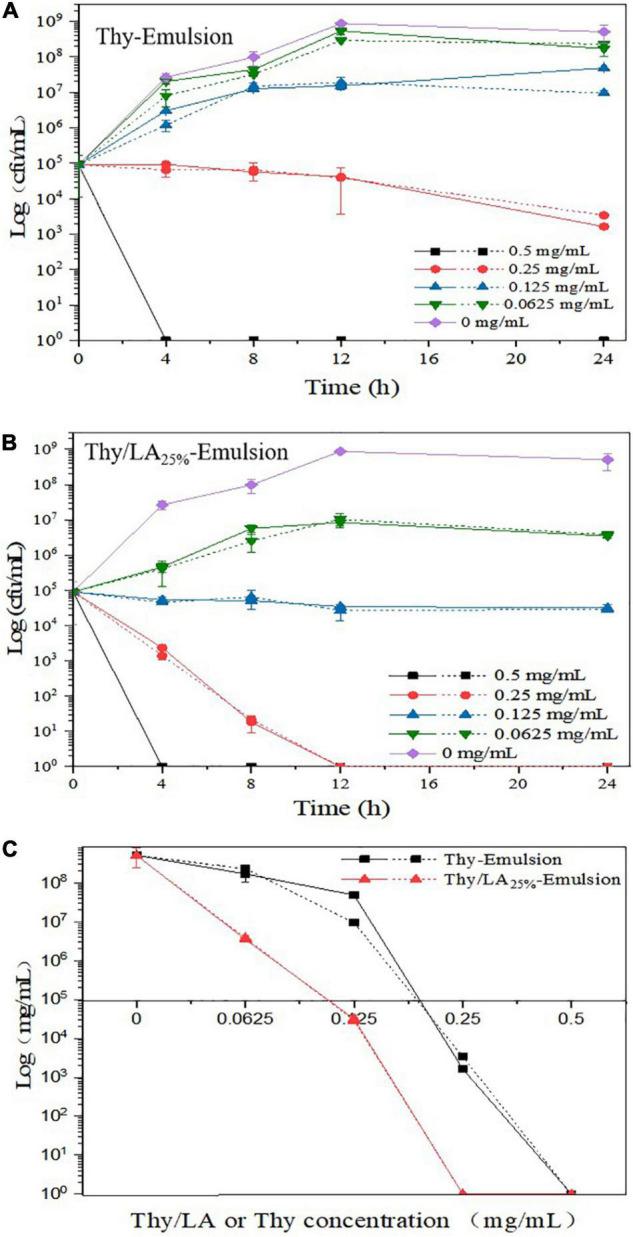
The growth curves of Thy-Emulsion **(A)** and Thy/LA_25%_-Emulsion **(B)** incubated for 24 h at 37°C and **(C)** the number of colonies formed in Thy-Emulsion and Thy/LA_25%_-Emulsion after 24 h (the concentration of Thy or Thy/LA ranged from 0.625 to 0.5 mg/mL). The solid line represents the 0th day, and the dashed line represents the 30th day. *n* = 6.

Antibacterial activity of two emulsions against *L. monocytogenes* was evaluated by using the zone of inhibition (Figure 6) and growth curves (Figure 7). As shown in Figure 5, the mean inhibition zone diameter of Thy/LA_25%_-Emulsion was 3.06 ± 0.12 cm, which was longer than the mean inhibition zone diameter of Thy-Emulsion (1.97 ± 0.06 cm). These results signified that Thy/LA_25%_-Emulsion exhibited a stronger antibacterial effect than Thy-Emulsion, indicating that the antibacterial effects of Thy and LA still demonstrate a synergistic effect in the emulsion delivery system (24). The concentration of Thy (Figure 7A) or Thy/LA (Figure 7B) in the oil phase ranged from 0.625 mg/ml to 0.5 mg/ml, and the higher the concentration of Thy or Thy/LA in the oil phase, the better the antibacterial effect of the emulsions possessed. After 24 h of cultivation, when the concentration of Thy or Thy/LA_25%_ was 0.125 mg/ml, the number of colonies in the Thy-Emulsion formed in the agar increased from approximately 9.0 × 10^4^ cfu/ml to 4.9 × 10^7^ cfu/ml, while the number of colonies in the Thy-Emulsion formed in the agar decreased from approximately 9.0 × 10^4^ cfu/ml to 3.2 × 10^4^ cfu/ml. The number of colonies in Thy-Emulsion formed in agar was approximately 10^4^ times more than it was in Thy/LA_25%_-Emulsion (Figure 7C). Thy/LA_25%_-Emulsion could completely inhibit the growth of *L. monocytogenes*, while Thy-Emulsion demonstrated weak antibacterial activity. When the concentration of emulsions was 0.25 mg/ml, the number of colonies in Thy-Emulsion formed in agar was approximately 10^3^ times more than it was in Thy/LA_25%_-Emulsion (Figure 7C). Thy/LA_25%_-Emulsion demonstrated a strong killing effect on *L. monocytogenes*, while Thy-Emulsion could only inhibit their growth. After 1-month of storage, the antibacterial activity of the two emulsions against *L. monocytogenes* was evaluated by using a growth curve (Figure 7), which is represented by a dashed line. The results showed that the antibacterial properties of Thy-Emulsion (Figure 7A) and Thy/LA_25%_-Emulsion (Figure 7B) did not decrease significantly after 1-month storage. These results indicated that Thy/LA_25%_-Emulsion demonstrated better antibacterial activity and physicochemical stability due to its long-term storage stability.

These results exhibited that the MIC values of Thy-Emulsion (0.5 mg/ml) were higher than those samples with Thy (0.25 mg/ml) alone, so did Thy/LA_25%_-Emulsion. These results were broadly in line with the findings of Chang et al. (32), where Thy emulsions were prepared as potential antimicrobial delivery systems and found that increasing the levels of ripening inhibitor in the oil phase reduced the antimicrobial efficacy of emulsions. This is because the partition of a lipophilic antibacterial agent between oil phase and aqueous phase hinges on their relative concentration and oil–water partition coefficient. After incorporating corn oil, the antibacterial agent will be more likely to partition into the oil phase of the emulsion, causing a decrease in the concentration of the antibacterial agent in the aqueous phase. Because microorganisms exist in the aqueous phase, the effective antibacterial concentration on the surface of microorganisms also decreases, thereby reducing the antibacterial effect (46, 47).

Above all, the incorporation of ripening inhibitor (corn oil) will reduce the antibacterial effect, but the antibacterial effect is still considerable due to the synergistic effect of LA and Thy. When the concentration of Thy was 0.0625 mg/ml (Figure 7C), the number of colonies in Thy-Emulsion formed in agar was approximately 1.7 × 10^8^ cfu/ml, while Thy/LA_25%_-Emulsion demonstrated a strong killing effect. In summary, compared with Thy-Emulsion, Thy/LA_25%_-Emulsion reduced the content of Thy and enhanced its antibacterial activity. At the same time, Thy/LA_25%_-Emulsion not only lessens the unique odor and irritation of Thy, but also conforms to the prevailing market in terms of economic benefits due to the low price of MCFs.

## Conclusion

We studied the antibacterial effects of LA addition on thymol-based emulsions. The incorporation of LA (25 and 50%) could improve the antibacterial activity of thymol-based emulsions against Gram-positive bacteria, especially *L. monocytogenes*. Compared with Thy-Emulsion, Thy/LA_25%_-Emulsion demonstrated a better antibacterial effect. Thy/LA_25%_-Emulsion exhibited a greater inhibition zone (3.06 ± 0.12 cm) than Thy-Emulsion (1.97 ± 0.06 cm). When a complete antibacterial effect was achieved against *L. monocytogenes*, the concentration of the antibacterial components (Thy) in the Thy-Emulsion was 0.5 mg/ml, while the concentration of the antibacterial components (Thy and LA) in the Thy/LA_25%_-Emulsion was 0.25 mg/ml. The concentration of Thy in the Thy/LA_25%_-Emulsion was 0.19 mg/ml, which lessens unique odor and irritation and saves cost by reducing the amount of Thy. Owing to the long-term storage stability, Thy/LA_25%_-Emulsion demonstrated the best antibacterial activity and physicochemical stability. This study may provide a useful and novel antibacterial measure for food and drugs.

## Data Availability Statement

The original contributions presented in the study are included in the article/supplementary material, further inquiries can be directed to the corresponding authors.

## Author Contributions

QC and SL: designed and conceived the study. QC, YZ, and XF: performed the experiments. QC: analyzed the data and drafted the manuscript. SL, ZH, SP, and WL: contributed to the writing of the manuscript. WL: provided the funding and resources. All authors revised and approved the submitted version of the manuscript.

## Conflict of Interest

ZH is employed by Jiangxi Danxia Biol Technol Co., Ltd. The remaining authors declare that the research was conducted in the absence of any commercial or financial relationships that could be construed as a potential conflict of interest.

## Publisher’s Note

All claims expressed in this article are solely those of the authors and do not necessarily represent those of their affiliated organizations, or those of the publisher, the editors and the reviewers. Any product that may be evaluated in this article, or claim that may be made by its manufacturer, is not guaranteed or endorsed by the publisher.
